# New Parvovirus in Child with Unexplained Diarrhea, Tunisia

**DOI:** 10.3201/eid2011.140428

**Published:** 2014-11

**Authors:** Tung G. Phan, Khira Sdiri-Loulizi, Mahjoub Aouni, Katia Ambert-Balay, Pierre Pothier, Xutao Deng, Eric Delwart

**Affiliations:** Blood Systems Research Institute, San Francisco, California, USA (T.G. Phan, X. Deng, E. Delwart);; University of California at San Francisco, San Francisco (T.G. Phan, E. Delwart);; University Hospital of Dijon, Dijon, France (K. Sdiri-Loulizi, K. Ambert-Balay, P. Pothier);; University of Monastir, Monastir, Tunisia (K. Sdiri-Loulizi, M. Aouni)

**Keywords:** parvovirus, diarrhea, feces, child, viruses, Tunisia, Tusavirus

## Abstract

A divergent parvovirus genome was the only eukaryotic viral sequence detected in feces of a Tunisian child with unexplained diarrhea. Tusavirus 1 shared 44% and 39% identity with the nonstructural protein 1 and viral protein 1, respectively, of the closest genome, Kilham rat parvovirus, indicating presence of a new human viral species in the *Protoparvovirus* genus.

Parvoviruses are small icosahedral viruses with linear single-stranded DNA genomes of ≈5 kb that are associated with a wide spectrum of illnesses in humans and animals. The subfamily *Parvovirinae,* which infects vertebrates, is currently classified into 8 genera, 5 of which contain human parvoviruses (*Dependoparvovirus*, *Erythroparvovirus*, *Bocaparvovirus*, *Tetraparvovirus*, and *Protoparvovirus*) (*1*). In 2012, bufaviruses 1 and 2 were sequenced from the feces of children with diarrhea from Burkina Faso and a child with nonpolio acute flaccid paralysis from Tunisia (*2*) and classified as founding members of the *primate protoparvovirus 1* species (*1*). Bufavirus DNA also was detected in diarrheal samples from adults in Finland and Holland (*3*,*4*) and a child from Bhutan (*5*). A related protoparvovirus was recently found in feces and serum from rhesus monkeys with simian AIDS in primate research centers (*6*). Here we describe the genome of a new parvovirus that we propose as prototype for a new species, *primate protoparvovirus 2*.

## The Study

Using metagenomic deep sequencing, we analyzed fecal samples from 180 infants and children ages 7 days–96 months (mean 18.7 months) in Tunisia who had unexplained diarrhea that tested negative for rotavirus, norovirus, astrovirus, sapovirus, adenovirus types 40 and 41, and Aichi virus by reverse transcription PCR (*7*). The University of California at San Francisco committee on human research approved the study.

The fecal supernatants were filtered through a 0.45-μm filter (Millipore, Darmstadt, Germany) to remove bacterium-sized particles, and the filtrates were digested with a mixture of DNases (Turbo DNase from Ambion, Carlsbad, CA, USA; Baseline-ZERO from Epicenter, Madison, WI, USA; and Benzonase from Novagen, San Diego, CA, USA) and RNase (Fermentas, Pittsburgh, PA, USA) to digest unprotected nucleic acids. Enriched viral nucleic acids (RNA and DNA) were then extracted and amplified by using ScriptSeq v2 RNA-Seq Library Preparation Kit (Epicenter) and analyzed in pools of 10 specimens in 2 Illumina MiSeq run of 250-bp end reads, yielding 20,693,619 unique sequences. We compared the Illumina sequences with the GenBank nonredundant protein databases using BLASTx (http://blast.ncbi.nlm.nih.gov/Blast.cgi).

Using a BLASTx E-score cutoff of 10^−5^, we identified, in decreasing frequency, sequences related to the mammalian viruses: sapovirus (120,177 reads), anelloviridae (14,841 reads), parechovirus (10,557 reads), norovirus (4,551 reads), enterovirus (3,857 reads), circoviridae (2,127 reads), group A rotavirus (839 reads), adeno-associated virus (812 reads), picobirnavirus (274 reads), bufavirus (168 reads), WU polyomavirus (136 reads), bocavirus (62 reads), adenovirus (58 reads), papillomavirus (22 reads), cosavirus (20 reads), group C rotavirus (17 reads), human astrovirus 1 (14 reads), salivirus (4 reads), and Aichi virus (2 reads). One pool showed a single read encoding a parvovirus-like protein segment with high levels of genetic similarity (BLASTx E-score of 5 × 10^−8^) to the nonstructural protein (NS) 1 of rat parvovirus (GenBank accession no. AFV67813).

The individual fecal sample within the pool containing this sequence was then identified by using PCR and underwent further deep sequencing as above, generating 11 more parovirus sequences. No other eukaryotic viral sequences were identified from 260,000 unique sequence reads from this patient. The near complete parvovirus genome was then acquired by filling genome gaps by PCR and amplifying 5′ and 3′ extremities using RACE (Rapid Amplification of cDNA Ends, Life Technologies). Amplicons were directly sequenced by primer walking. We named this virus Tusavirus 1 for Tunisian stool-associated parvovirus.

A nearly complete 4,424-bp genome (Tusavirus 1, GenBank accession no. KJ495710) was successfully acquired with partial 5′ untranslated region (243 bp), complete NS1 open reading frame (625 aa), complete viral protein (VP) 1 open reading frame (715 aa), and a partial 3′ untranslated region (68 bp). Tusavirus has a potential upstream start codon MSS in a weaker Kozak consensus sequence than MAQ (Figure, panel A), which we selected as the start codon. The Walker loop ^396^GPATTGKS^403^ [GXXXXGK(T/S)], which is an ATP- or GTP-binding motif, was found in the NS1. We also identified 2 conserved replication initiator motifs ^127^EFHIHVLLW^135^ and ^187^VLQYKHSQTR^196^. Potential splicing signals to express VP1 were identified on the basis of alignments to other protoparvoviruses and classic RNA splicing motifs (Figure, panel A). The phospholipase A_2_ (PLA2) motif was identified in VP1 N-termini with expected calcium-binding site and catalytic residues. The methionine codon of VP2 was located upstream of glycine-rich sequence (GGGARAGGVG). An unusual serine-rich sequence (SSSDSGPSSS) was also seen near VP1 N-termini.

**Figure:**
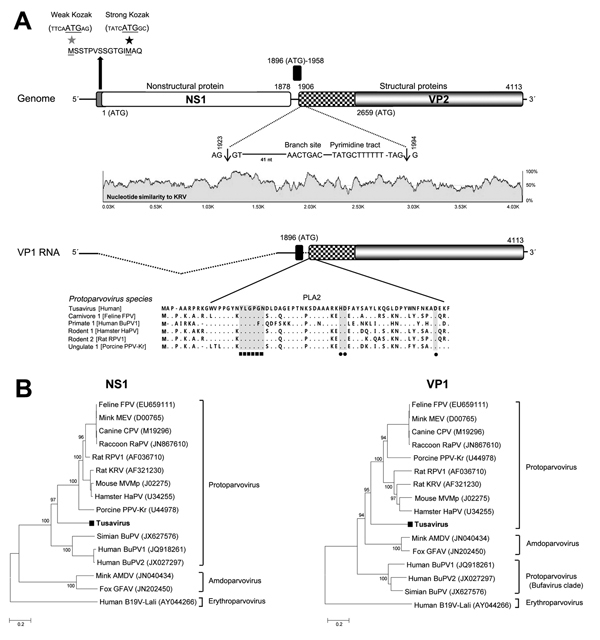
New parvovirus genome and phylogeny. A) Organization of the Tusavirus genome. Theoretical splicing for expression of viral protein (VP) 1 is shown. The alignment of the PLA2 regions of representatives of 5 protoparvovirus species show the calcium-binding region and catalytic residues in Tusavirus. Pairwise sliding window of percentage nucleotide similarity of Tusavirus aligns with the genetically closest Kilham rat parvovirus. B) Phylogenetic trees generated with nonstructural protein (NS) 1 and VP1 of Tusavirus and of the 5 International Committee on Taxonomy of Viruses–designated species in the *Protoparvovirus* genus. Scale bar indicatesamino acid substitutions per site. FPV, feline parvovirus; MEV, mink enteritis virus; CPV, canine parvovirus; RaPV, raccoon parvovirus; RPV1, rat parvovirus 1; MVMp, minute virus of mice, prototype; HaPV, hamster parvovirus; PPV-Kr, porcine parvovirus Kresse; Simian BuPV, Simian bufavirus; BuPV1, bufavirus 1; BuPV2, bufavirus 2; AMDV, Aleutian mink disease virus; GFAV, gray fox amdovirus; B19V-Lali, human parvovirus B19-Lali. Bootstrap values (based on 100 replicates) for each node are given if >70%.

Protein sequence alignments were made by using ClustalX version 2.0.3 (http://www.clustal.org) with the default settings; a phylogenetic tree with 100 bootstrap resamples of the alignment datasets was generated by using the neighbor-joining method based on the Jones-Taylor-Thornton matrix-based model in MEGA5 (http://www.megasoftware.net). Bootstrap values for each node are given if >70%. Resulting trees were examined for consistency with published phylogenetic trees.

Phylogenetic analysis showed that Tusavirus 1 was distinct from known members of the *Protoparvovirus* genus (Figure, panel B). NS1 shared the highest identity to the Kilham rat parvovirus (44%). VP1 and VP2 shared identities of 39% and 37%, respectively, to those of Kilham rat parvovirus. According to the International Committee on Taxonomy of Viruses, the members of the same parvovirus genus should share >30% and members of the same species >85% aa identity in NS1. The *Protoparvovirus* genus currently comprises species infecting carnivores, rodents, pigs, and humans (*1*). Tusavirus 1 is proposed as prototype for *primate protoparvovirus 2* species that would join bufaviruses as human viruses in this genus.

We used a nested PCR targeting NS1 to determine the prevalence of this virus in the 180 Tunisia diarrhea samples. Primers Tusa-F1 (5′-GAAGAAGCTGGAAACTGTGGTCA-3′) and Tusa-R1 (5′-CTCGTCTTTCTCCCAGGCATCT-3′) were used for the first round of PCR, and primers Tusa-F2 (5′-ATTGCTCCAACACCAGTCATCA-3′) and Tusa-R2 (5′-TCTGGTCTGGTCCAATCTTCTTC-3′) for the second round of PCR. The PCR conditions were: 95°C for 5 min, 35 cycles 95°C for 30 s, 52°C or 51°C (for the first or second round, respectively) for 30 s, and 72°C for 1 min, a final extension at 72°C for 10 min. No samples except the one initially detected by deep sequencing were PCR positive, yielding a low prevalence of 0.56% (1/180) in this Tunisian population.

## Conclusions

We detected fecal shedding of a previously uncharacterized parvovirus in a child with unexplained diarrhea. The 18-month-old girl showed twice daily liquid and greenish feces over 3 days but no fever (37.4°C), vomiting, or dehydration. To identify other viral infections in this patient, the Tusavirus-positive sample was individually analyzed by the same metagenomics method. No other mammalian virus was detected, suggesting a possible role for Tusavirus 1 in this patient’s gastrointestinal illness, although the lack of testing for pathogenic bacteria and parasites does not enable us to exclude these alternative explanations. Wider geographic sampling of human samples, including case–control PCR studies of unexplained diarrhea and serologic tests, are needed to define the prevalence and disease association of this new parvovirus species in different age groups and populations.
